# The Hippo Signaling Transducer TAZ Regulates Mammary Gland Morphogenesis and Carcinogen-induced Mammary Tumorigenesis

**DOI:** 10.1038/s41598-018-24712-5

**Published:** 2018-04-24

**Authors:** Kayla E. Denson, Ashley L. Mussell, He Shen, Alexander Truskinovsky, Nuo Yang, Natesh Parashurama, Yanmin Chen, Costa Frangou, Fajun Yang, Jianmin Zhang

**Affiliations:** 1 Department of Cancer Genetics & Genomics, Roswell Park Cancer Institute, Buffalo, NY 14263 USA; 2 Department of Pathology, Roswell Park Cancer Institute, Buffalo, NY 14263 USA; 3 Department of Anesthesiology, University at Buffalo, The State University of New York, NY, 14214 USA; 4 Department of Chemical & Biological Engineering, University at Buffalo, The State University of New York, NY, 14214 USA; 5 Harvard TH Chan School of Public Health, Molecular and Integrative Physiological Sciences, 665 Huntington Avenue, Boston, MA 02115 USA; 6 Departments of Medicine, Diabetes Research Center, Albert Einstein College of Medicine, Bronx, NY 10461 USA

## Abstract

Hippo signaling pathway is an evolutionarily conserved pathway that controls organ size by regulating cell proliferation, apoptosis and stem cell self-renewal. TAZ (transcriptional coactivator with the PDZ-binding motif) is a key downstream effector of the mammalian Hippo pathway. Here, using a transgenic mouse model with mammary-gland-specific expression of constitutively active TAZ, we found that TAZ induction in mammary epithelial cells was associated with an increase in mammary glandular size, which probably resulted from adipocyte hypertrophy. Consistent with its known oncogenic potential, we observed tumor formation in TAZ transgenic mice after administration of the carcinogen 7,12-dimethylbenzanthracene (DMBA) and demonstrated that tumorigenesis was reliant on the presence of TAZ. Our findings establish a previously unknown roles of TAZ in regulating both mammary gland morphogenesis as well as carcinogen-induced mammary tumor formation.

## Introduction

The Hippo signaling pathway was first identified in *Drosophila* through genetic screens and regulates organ size by modulating cell growth, proliferation and apoptosis^1–3^. A majority of the Hippo pathway components are highly conserved from *Drosophila* to mammals, and dysregulation of this pathway is frequently observed in cancers^4–6^. One cascade involves the upstream Hippo pathway component STE20-like kinase 1/2 (MST1/2), which acts in a complex with its regulatory protein salvador 1 (SAV1) to phosphorylate and activate large tumor suppressor kinase 1/2 (LATS1/2) and its regulatory protein MOB kinase activator 1 A (MOB1A). This, in turn, phosphorylates and inactivates the transcriptional coactivators, Yes-associated protein (YAP) and transcriptional coactivator with the PDZ-binding motif (TAZ). Once YAP/TAZ translocate into the nucleus, they induce the expression of cell-proliferative and anti-apoptotic genes, mainly through the interactions with transcription factors such as TEA domain family members (TEADs)^3^. During the past decade, the complexity of YAP/TAZ regulation has been increasingly revealed. For example, the G protein-coupled receptors (GPCRs) and leukemia inhibitory factor receptor (LIFR) are found to be associated with the activation of LATS kinases^7,8^. Furthermore, YAP/TAZ are also directly regulated by the extracellular matrix (ECM)^9^, mechanotransduction^10,11^, actin cytoskeleton and Rho GTPases^11,12^.

The mammary gland is a unique glandular organ that reaches full development only after birth^13^. The three main stages of mammary gland development are embryonic, pubertal and adult. Hormones and growth factors play pivotal roles in these different stages of mammary development. Moreover, dysregulation of mammary gland development is also implicated in breast cancer^14^. The mammary gland consists of a variety of cell types: luminal and basal epithelial cells that form the mammary gland ducts, adipocytes that constitute the fat pad, vascular endothelial cells that form the blood vessels, stromal cells including fibroblasts as well as numerous immune cells distributed in the gland^13–15^. Loss of function of the Hippo pathway components, such as Sav1, Lats1, Yap and Taz, has been studied in the mouse mammary gland development^16^. For example, it has been reported that Sav1 and Yap are dispensable in virgin mammary gland development but required specifically during pregnancy, while loss of Sav1 and Yap affect the mammary cell differentiation and survival^17^. Also, Taz-deficient mouse mammary glands are normal in pubescent virgin (5 to 8 weeks old), but the number and complexity of mammary gland branches are reduced in the post-pubertal virgin (16 weeks old) stage^18^. Surprisingly, there has been no study on the effect of Hippo pathway components and how it controls other key features of mammary gland biology such as, organ size and malignant transformation.

We addressed this gap in knowledge by developing a transgenic mouse model with mammary-gland specific TAZ activation. We found that over-expression of TAZ in mammary epithelial cells induced advanced mammary gland development in the early puberty stage. In addition, activation of TAZ enlarged mammary gland size by increasing the fat mass. Lastly, TAZ activation induced massive tumor formation in response to carcinogen (DMBA) treatment. Collectively, our study demonstrates an important role of TAZ in mammary gland development and more intriguingly, an unexpected effect on mammary adipose tissue.

## Results

### TAZ expression in mammary epithelial cells advances mammary gland development

**Figure 1:**
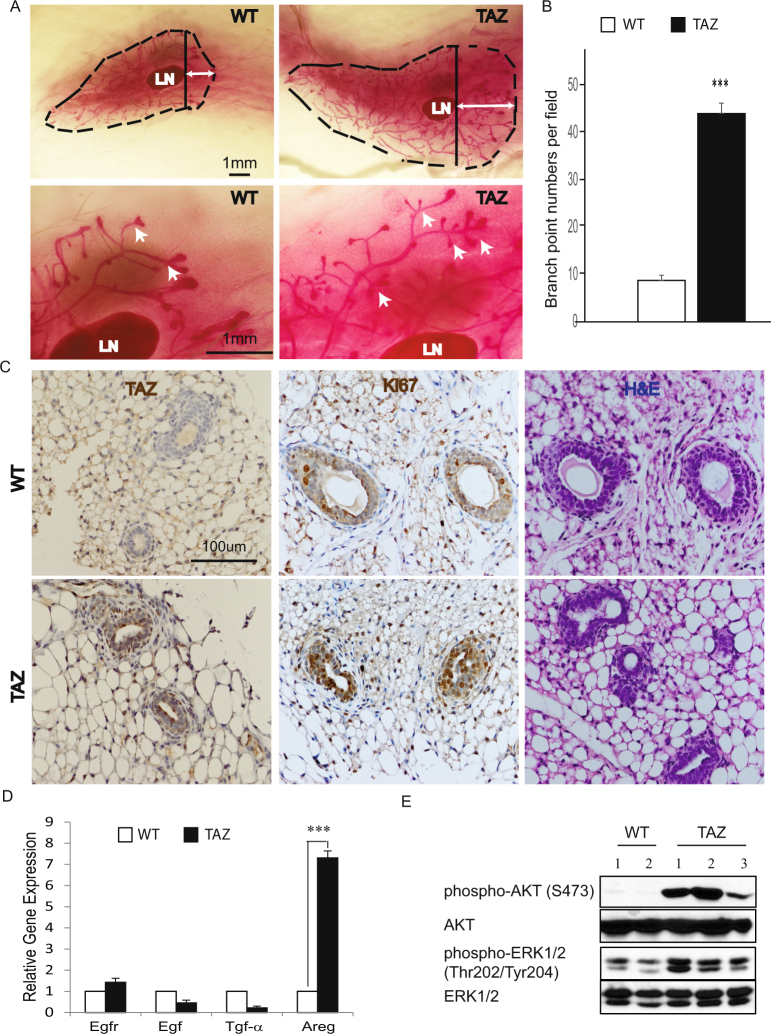
TAZ accelerates the branching morphogenesis of mammary gland. (**A**) Representative images of whole-mount staining of wild-type (WT) or MMTV-rtTA/TRE-TAZ mammary gland at 4 weeks. LN: lymph node; broken line: outline of mammary gland; solid line: start point of mammary gland tree invasion beyond the LN; arrow: advanced branching and TEB invasion. Similar findings observed in n = 6 mice per group (Scale bar = 1 mm). (**B**) Quantitation of WT or MMTV-rtTA/TRE-TAZ mammary gland branch numbers at 4 weeks. n = 3 mice per group; error bars represent SD; ***p < 0.001 by two-tailed student’s t-test. (**C**) H&E, immunohistochemistry staining for TAZ and Ki67 in 4-week WT or MMTV-rtTA/TRE-TAZ mammary gland. (Scale bar = 100 µm). (**D**) Real-time qPCR measured expression levels of Egf, Areg, Tgf-α and Egfr of 4-week WT or MMTV-rtTA/TRE-TAZ mammary gland. n = 3 mice per group; error bars represent standard deviation (SD); ***p < 0.001 by two-tailed student’s t-test. (**E**) Immunoblot of AKT, phospho-AKT (S473), ERK1/2 and phospho-ERK1/2 (Thr202/Tyr204) expression in WT or MMTV-rtTA/TRE-TAZ mammary gland. n = 2 WT mice; n = 3 MMTV-rtTA/TRE-TAZ mice.

Both YAP and TAZ have been shown to be involved in breast cancer stem cell regulation^19–22^ as well as drug resistance in breast cancer (BC)^23,24^. To detect the effect of TAZ activation on mammary gland development, we first developed an inducible transgenic mouse model with an overexpression of a constitutively active form of human TAZ (TAZ^4SA^: S66, S89, S117 and S311 substituted with alanine)^25,26^ under the control of a tetracycline response element (TRE) (Clontech) (Figure S1A). The Tet-ON and-OFF systems are one of the most powerful tools for regulated transgene expression *in vivo*^27,28^. To confirm TAZ expression in response to doxycycline (dox), we established a TRE-TAZ^4SA^ transgenic mouse embryonic fibroblasts (MEF) cell line, and retrovirally transduced them with the pCMV-Tet3G vector. We found that TAZ was highly expressed in response to the dox induction (Data not shown). Next, to generate the mammary gland-specific TAZ^4SA^ expressing mice, we crossed our TRE-TAZ^4SA^ mice with the MMTV-rtTA mice purchased from the Jackson Laboratory^29^. To validate TAZ induction in the mammary gland in response to dox, we performed immunoblot analysis as well as immunohistochemical (IHC) staining using mammary glands harvested from the MMTV-rtTA/TRE-TAZ^4SA^ bi-transgenic mice fed with the dox-containing or control chow. These analyses showed that TAZ was indeed highly induced by dox (Figure S1B & Fig. 1C).

The mammary gland undergoes most of its development after birth, under the influence of female reproductive hormones^30^. During embryogenesis, a rudimentary ductal system develops that grows isometrically with the rest of the body in the first weeks of life. At the onset of puberty, when the ovaries start to secrete estrogen, the ducts extend from the nipple area into the mammary fat pad. The tips of the ducts enlarge to form club-shaped structures called terminal end buds (TEBs), which contain highly proliferative cells and mammary stem cells^31,32^. To our great interest, the advanced branching complexity of TEBs was markedly elongated in TAZ mice in the presence of dox. Notably, these structures extended and invaded to a greater degree into the mammary fat pad in contrast to control TEBs at 4 weeks (Fig. 1A). TAZ transgenic mice also developed more secondary or tertiary mammary gland branches as well as increased branch point numbers compared to the control mice (Fig. 1A–B). To examine whether TAZ activation altered the proliferation of mammary epithelial cells, we performed IHC and observed strong staining of Ki67 in the TAZ mice compared to control (Fig. 1C). Previously, we have demonstrated that activation of TAZ increases the expression of amphiregulin (AREG), a ligand of the epidermal growth factor receptor (EGFR), in human mammary epithelial MCF10A cells^33^. Consistent with this observation, here we found prominent *Areg* expression as well as the AKT and ERK activation in the TAZ mice (Fig. 1D–E). In summary, our data revealed that activation of TAZ drove advanced mammary gland development at a pre-pubertal stage and that there was also a concurrent activation of the AKT and ERK pathway.

### Tissue-specific induction of TAZ increased mammary gland size and weight

**Figure 2:**
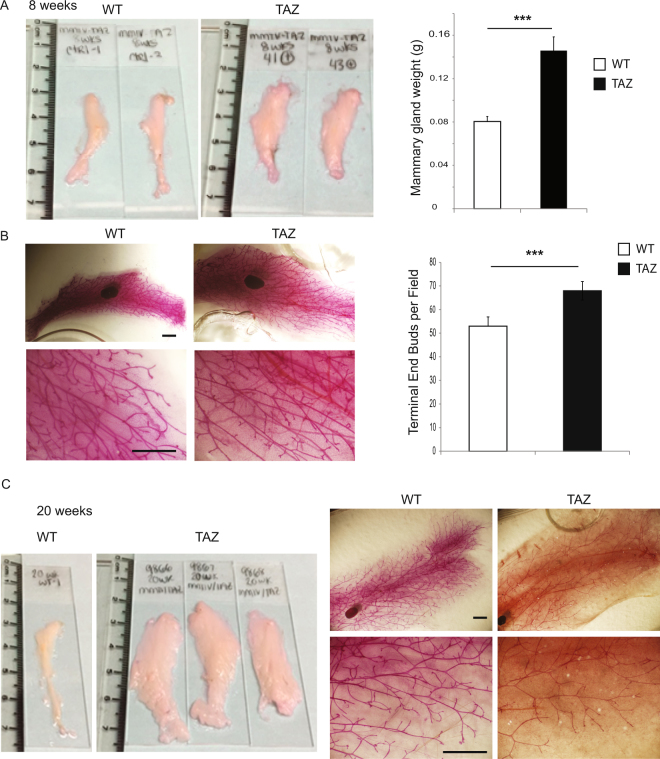
TAZ activation increases the mammary gland size. (**A**) Representative images of mammary gland and weight quantification of 8-week WT or MMTV-rtTA/TRE-TAZ mice. n = 6 mice per group; error bars represent SD; ***p < 0.001 by two-tailed student’s t-test. (**B**) Representative images of mammary gland whole-mount staining and quantification of terminal end buds of WT or MMTV-rtTA/TRE-TAZ mice. n = 3 mice per group (Scale bar = 1 mm). (**C**) Representative images of mammary gland and whole-mount staining of 20-week WT or MMTV-rtTA/TRE-TAZ mice. n = 5 mice per group.

Intriguingly, we observed increased size and weight of the mammary glands in the TAZ mice as compared to the control at eight weeks (Fig. 2A). The mammary gland ductal trees occupied the entire enlarged gland with increased gland area and branch length of the mammary ductal trees (Fig. 2B). Furthermore, strong activation of AKT and ERK signaling has also been observed in TAZ mice at this developmental stage (Figure S1C). In addition, we observed the continuously enlarging mammary glands in the TAZ mice up to 20 weeks (Fig. 2C). Taken together, overexpression of TAZ in mammary epithelial cells increased mammary gland size in pubescent and post-pubertal virgin mice.

### Constitutively active TAZ results in mammary gland adipocyte hypertrophy

**Figure 3:**
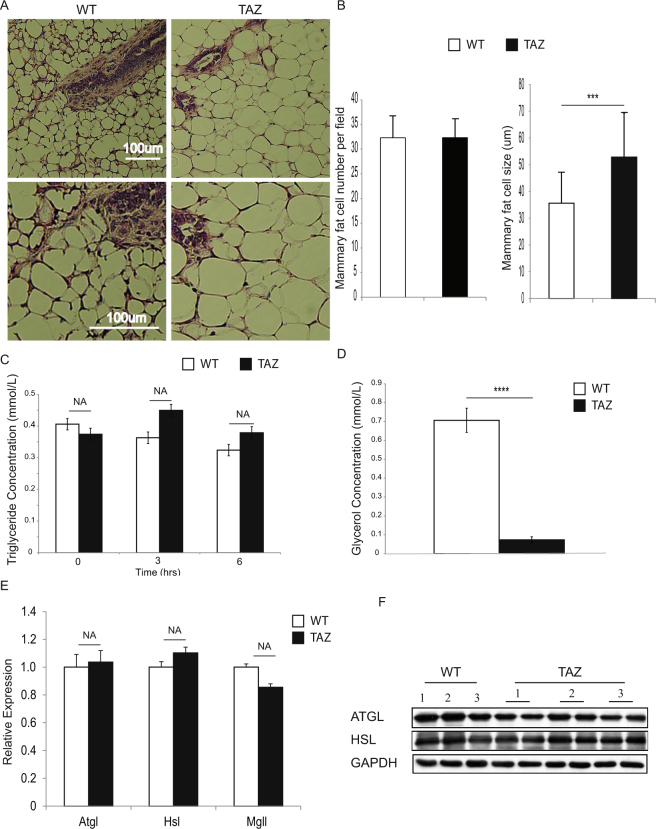
TAZ activation increases fat storage and inhibits lipolysis of the mammary gland adipocytes. (**A**) H&E staining of mammary gland for 8-week WT or MMTV-rtTA/TRE-TAZ mice. (Scale bar = 100 µm). (**B**) Quantification of the number and size of mammary gland adipocytes in 8-week WT or MMTV-rtTA/TRE-TAZ mice. n = 3 mice per group; 5 fields were counted per group; error bars represent SD; ***p < 0.001 by two-tailed student’s t-test. (**C**) Measurement of the circulation of triglyceride. n = 3 mice per group; NA, no significant difference by two-tailed student’s t-test. (**D**) Measurement of *ex vivo* mammary gland lipolysis. n = 3 mice per group; error bars represent SD; ***p < 0.001 by two-tailed student’s t-test. (**E**) Real-time qPCR measured expression levels of *Atgl*, *Hsl* and *Mgl* of 8-week WT or MMTV-rtTA/TRE-TAZ mammary gland. n = 3 mice per group; NA, no significant difference by two-tailed student’s t-test. (**F**) Immunoblot of ATGL and HSL expression in WT or MMTV-rtTA/TRE-TAZ mammary gland. n = 3 WT mice 3 mammary glands; n = 3 MMTV-rtTA/TRE-TAZ mice 6 mammary glands. GAPDH was used as loading control.

Adipose tissue plays a critical role in homeostasis across all states in mammals, including during mammary gland development^34^. Expanding the fat mass requires either increased adipocyte size (hypertrophy) or increased adipocyte number (hyperplasia)^35^. To determine whether TAZ induced enlargement of the mammary gland through adipocyte hypertrophy or hyperplasia, we analyzed both size and number at eight weeks. We found increased mammary gland adipocyte size but not cell number in the TAZ mice (Fig. 3A,B), indicating that the transgenic TAZ overexpression in mammary epithelial cells induced mammary adipocyte hypertrophy. Such hypertrophy could result from either increased abnormal lipid synthesis (lipogenesis) or decreased lipid release (lipolysis). To begin evaluation of the underlying mechanism(s), we measured the circulating triglyceride concentration in response to intralipid injection at different time points after short-term animal fasting. No significant difference was observed in the circulating triglyceride levels between WT and TAZ mice (Fig. 3C). To further determine whether there was any difference in lipolysis, we isolated mammary glands from either WT or TAZ mice and performed *ex vivo* lipolysis analysis. Notably, we detected a dramatic reduction of lipolysis in the TAZ mammary gland (Fig. 3D), indicating that the enlarged TAZ mammary gland may result from decrease in mammary gland adipocyte lipolysis. Adipose triglyceride lipase (ATGL), hormone-sensitive lipase (HSL) and monoacylglycerol lipase (MGL) are the main enzymes catalyzing lipolysis in the white adipose tissue (WAT)^36,37^. To detect whether the expression of these enzymes was changed in TAZ mice, we harvested mammary glands from WT or TAZ mice and performed qRT-PCR and immunoblot. We did not detect significant mRNA alterations of these enzymes (Fig. 3E), but observed a modest reduction of ATGL protein level in TAZ mice (Fig. 3F). No difference in HSL protein levels was detected (Fig. 3F). Our results suggest that TAZ activation leads to reduction of mammary gland adipocyte lipolysis in part through down-regulation of ATGL proteins.

### TAZ enhances tumor formation in response to carcinogen (DMBA) treatment

**Figure 4:**
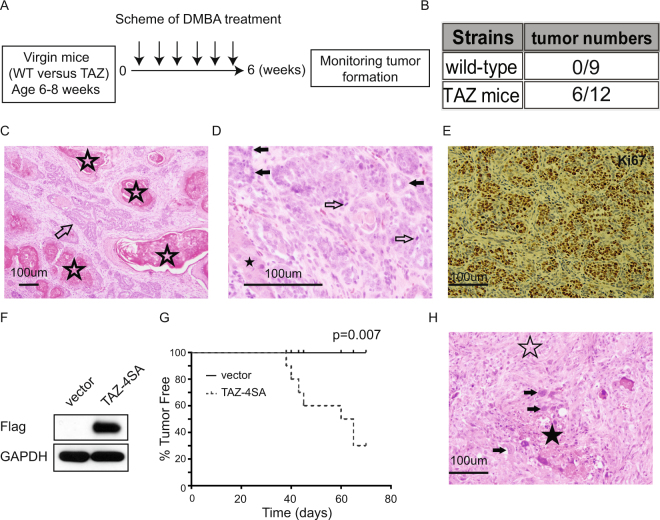
Carcinogen DMBA treatment leads to mammary tumor formation in the TAZ transgenic mice. (**A**) Schematic illustration of protocol for testing DMBA administration. (**B**) Incidence of tumor formation in WT or MMTV-rtTA/TRE-TAZ mice in response to DMBA treatment. (**C**) DMBA-induced adenosquamous carcinoma, with both glandular (arrow) and squamous (stars) differentiation (H&E, original magnification x100). (**D**) Higher magnification of DMBA-induced tumor showing moderately differentiated adenosquamous carcinoma with glandular (filled arrows) and squamous (star) differentiation and scattered mitotic figures (outline arrows). (H&E, original magnification x400). (**E**) Immunohistochemistry staining of Ki67 in DMBA-induced mammary gland tumor. (**F**) Immunoblot of TAZ expression. GAPDH was used as loading control. (**G**) Incidence of tumor formation from subcutaneously injected tumor-derived cells. n = 6 mice per group (**H**) Subcutaneously grown, poorly differentiated malignant neoplasm with features of carcinosarcoma, showing predominant spindle cells (outline star), areas of necrosis (filled star) and numerous cells with markedly enlarged, atypical nuclei (arrows). No glandular or squamous differentiation detectable. (H&E, original magnification x200).

Although overexpression of TAZ led to mammary gland enlargement, no tumor formation was observed in the TAZ transgenic mice monitored up to 64 weeks of age. Thus, to investigate the oncogenic potential of constitutively active Taz in mammary tumorigenesis, we treated wild-type or TAZ transgenic mice with the carcinogen 7,12-dimethylbenz(a)anthracene (DMBA). Strikingly, mammary tumors were then observed in 50% (n = 12) of the TAZ mice as compared to 0% (n = 9) of the control group after eight weeks of the carcinogen treatment (Fig. 4A–B). Further histological analysis revealed that the majority of induced mammary tumors were moderately differentiated adenosquamous carcinoma (Fig. 4C–D). Immunohistochemical staining of Ki67 showed these tumors to be hyperproliferative (Fig. 4E). Finally, to test whether the DMBA-induced tumor formation was dependent on TAZ expression, we isolated DMBA-induced tumor-derived isogenic cells and transduced them with the TAZ-^4SA^ or control vector (Fig. 4F). These cells were then subcutaneously injected into the severe combined immunodeficiency (SCID) mice. As summarized in Fig. 4G, we observed the formation of large tumors but only in animals that received TAZ-transduced cells (Fig. 4G). Correspondingly, histological analysis revealed poorly differentiated malignant neoplasms with features of carcinosarcoma (Fig. 4H), with spindle cell morphology as well as areas of necrosis, high-grade nuclear atypia and frequent markedly enlarged, atypical nuclei. Together, the results demonstrate that TAZ overexpression enhanced mammary tumorigenesis in response to carcinogen treatment.

## Discussion

The Hippo signaling pathway was initially identified in *Drosophila* and subsequently shown to be a conserved regulator of organ size, tissue regeneration and stem cell biology^1^. Liver-specific knockout of the Hippo pathway upstream proteins Mst1/2, WW45 or overexpression of YAP in transgenic mice leads to increased liver size and hepatocellular carcinomas^38–40^. In contrast, mammary gland-specific knockout *Sav1* (WW45) or overexpression of YAP did not affect mammary gland development at the virgin stage, but instead caused defects in the terminal differentiation of secretory cells during lactation^17^. Both Chen and Skibinski *et al*. showed that mice with mammary-gland-specific deficiency of *Yap* or *Taz* still exhibited normal mammary glands at the pubescent virgin stage^17,18^, but the number and complexity of mammary gland branches were reduced in post-pubertal virgin *Taz* mice^18^ whereas *Yap* was indispensable for the mammary epithelial cell survival during pregnancy^17^. Both of these two reports indicated an essential role of *Yap/Taz* in the later stages of mammary gland development. On the other hand, our data demonstrate that TAZ activation also can accelerate mammary gland branching at the pre-pubertal stage of virgin mice, an effect which may partially result from the activation of PI3K and MAPK signaling.

TAZ was initially identified as a 14-3-3 binding protein^41^. TAZ also interacts with the transcription factor RUNX2 and drives the development of the osteoblast lineage while coordinately repressing PPARγ-dependent gene transcription, which also identifies its importance in the adipocyte lineage commitment^42^. Recently, Matsumoto *et al*. reported the interplay between ABL and TAZ creates an amplification loop that stabilizes and activates a RUNX2-TAZ transcriptional complex needed to “lock in” mesenchymal development toward the osteoblast lineage^43^. Meanwhile, TAZ can promote adipogenesis through the inhibition of canonical Wnt signaling^44^. Last but not least, in *Drosophila* the loss of function of Hpo (MST1/2) or overexpression of Yki (YAP/TAZ) in fat bodies result in weight gain and greater fat storage^45^. Consistent with the aforementioned studies, we show here that TAZ increased fat storage in the mammary glands through a non-autonomous effect on lipolysis in the mammary gland adipocytes. Lipolysis is the process of hydrolyzing triacylglycerol (TG) to fatty acids (FAs) and glycerol *via* the sequential actions of ATGL, HSL and MGL^46^. Although the mRNA levels of these enzymes were not affected, TAZ overexpression slightly reduced ATGL but not HSL protein levels. The mechanism(s) of such changes are currently unclear and requires further investigation. Nonetheless, the small reduction of ATGL proteins can only partially explain the overall reduction of lipolysis rate. It is likely the activities of ATGL and HSL are also reduced in TAZ mice. Further analysis of ATGL and HSL modifications and enzymatic activities is required to understand dysfunction of lipolysis in TAZ mice. Insulin is a well-known key factor to inhibit lipolysis^47^. Of particular note, it has been reported that the insulin/IGF signaling (IIS) pathway and the Hpo/Yki signaling pathway are intricately interlinked in normal development as well as in cancer^48–50^. It will be very interesting to further investigate whether TAZ alters the mammary gland adipocyte lipolysis through the insulin/IGF signaling.

We did not detect mammary tumor formation in the TAZ transgenic mice. Overexpression of YAP alone is insufficient to drive oncogenic growth in the normal mammary epithelia^17^. It is thus suggested that YAP/TAZ-initiated mammary gland tumorigenesis may require cooperating genetic lesions. Indeed, the *Yap-*containing amplicon was initially identified in the *Brca1/p53*-deficient mice^24^ and was also recently shown in the tumors generated from the luminal mammary epithelial cells with loss-of-function of p53^51^. Moreover, conditional knockout of YAP in the mammary gland increases the latency and reduces the growth of mammary tumors arising in the MMTV-PyMT mice^17^. We demonstrate here that the TAZ transgenic mice were susceptible to the DMBA carcinogen treatment. Many studies have revealed that the Hippo signaling is dysregulated in cancer^4,52,53^. Particularly in breast cancer, high YAP/TAZ activity has been correlated with cancer stemness, resistance to chemotherapy, high-grade histology, poor patient outcomes and metastasis^53^. Our study illustrates how the transgenic model we have developed can be used to understand mammary gland development and TAZ-driven tumorigenesis programs. More in-depth knowledge of TAZ and the Hippo signaling pathway is likely to inform future BC patient prognosis and therapy.

## Methods

### Transgenic mouse strains

The human-TAZ^4SA^ was cloned into the pTRE-Tight vector (Clontech, CA). Pronuclear injection of linearized pTRE-TAZ^4SA^ transgene was performed at the Targeting and Transgenic Core Facility of RPCI. The pTRE-TAZ^4SA^ transgenic mice were genotyped by a PCR primer set (in the 5′ to 3′ direction) - Forward primer: GCTCGTTTAGTGAACCGT and Reverse primer: TGTGGTGATTTTTTCTATGTG. The pTRE-TAZ^4SA^ transgenic mice were maintained in a C57BL6 background. The MMTV-rtTA strain (B6; SJL-Tg (MMTV-rtTA)4-1 Jek/J) was purchased from The Jackson Laboratory^29^. The MMTV-rtTA transgenic mice were genotyped by a PCR primer set (5′ to 3′) - Forward primer: CTGGTCATCATCCTGCCTTT and Reverse primer: GGCGAGTTTACGGGTTGTTA. For TAZ^4SA^ induction, transgenic and non-transgenic littermates were fed with Doxycycline-containing chow (200 mg/kg; Bioserve, NJ). The care and use of animals were performed under the rules provided by the Declaration of Helsinki and approved by the Institutional Animal Care and Use Committee of the Roswell Park Cancer Institute (Buffalo, NY).

### Mammary gland whole-mount staining

Mammary glands #4 were removed and spread out on a slide. The glands were allowed to air dry to the slide and then fixed overnight in Carnoy’s fixative (75% EtOH + 25% acetic acid). Glands were then washed in 70% EtOH for 15 minutes and rinsed in dH2O for 5 minutes. They were stained in Alum Carmine at least overnight (until lymphoid tissue is stained). After staining, glands were washed for 15 minutes each in 70%, 95%, and 100% EtOH, and then in acetone for 1 hour. The glands were cleared in xylene overnight and cover-slipped with Permount.

### Skeleton analysis of mammary gland branching

Analysis performed with the assistance of Natesh Parashurama (SUNY-Buffalo) as previously described^54^. For each whole-mount image, to convert ductal structures to skeletons in ImageJ, ducts were outlined, filled, and the entire image was converted to a binary image. The image was then processed using the “Skeletonize” function and the final skeletonized image in each field of view was analyzed by the “Analyze Skeleton” tool. This image identifies all regions of interest (ROIs) and calculates several metrics, including area, number of junctions, number of junction voxels, number of branches, average branch length and slab voxels.

### *Ex vivo* lipolysis assay in mammary gland adipose tissue

Mammary fat pads were surgically removed from 16-week-old MMTV-rtTA/TRE-TAZ^4SA^ or wild-type mice and washed with ice-cold PBS. Mammary Fat pads (n = 6) were pre-incubated for 1 h in 140 µl of DMEM (Life Technologies, MA) containing 2% fatty acid-free serum albumin (Sigma-Aldrich, MO). Subsequently, mammary fat pads were incubated in 250 ul of KRH buffer (125 mM NaCl, 5 mM KCl, 1.8 mM CaCl_2_, 2.6 mM MgSO_4_, 5 mM HEPES, pH 7.2) plus 2% fatty acid free BSA (Sigma-Aldrich, MO) for 2 h at 37 °C. Free glycerol content was quantified for each sample in the medium using the Free Glycerol Determination Kit (Sigma-Aldrich, MO). Glycerol release from each sample was normalized to the weight of each mammary fat pad.

### Lipid clearance Assay

Sixteen-week-old MMTV-rtTA/TRE-TAZ^4SA^ transgene or wild-type mice were fasted overnight. Twenty percent Intralipid was then injected into the mice by oral gavage. Blood samples were harvested at T = 0, 3 and 6 hours after injection. Serum triglycerides were measured by the Infinity reagent (Thermo Fisher Scientific, MA).

### DMBA treatment

The carcinogen 7,12-dimethylbenz[a]anthracene (DMBA; Sigma-Aldrich, MO) was dissolved at 10 mg/kg in vegetable oil. Starting at 6 weeks of age, wild-type or MMTV-rtTA/TRE-TAZ^4SA^ transgenic mice were treated with DMBA by oral gavage once a week, for consecutive six weeks. Mice were checked for mammary tumors periodically and sacrificed after tumor discovery.

### Quantitative real-time PCR (qRT-PCR)

Total RNA was extracted from the mammary glands of 4, 8-week-old wild-type or MMTV-rtTA/TRE-TAZ^4SA^ mice using the Trizol Reagent (Life Technologies, MA) according to the manufacturer’s protocol. cDNA synthesis and quantitative real-time PCR was performed as previously described^55^. Gapdh was used as the internal control. The primer sequences were as follows:

Egf-F: 5′-GTCTCAGGGAGAAATCAGTCAC-3′

Egf-R:5′-GCTGTGACGCTGAGTATGCTAA-3′

Egfr-F: 5′-TCTTCAAGGATGTGAAGTGTG-3′

Egfr-R: 5′-TGTACGCTTTCGAACAATGT-3′

Tgf-α-F: 5′-GTGGCTGCCAGCCAGAAGAAGC-3′

Tgf-α-R: 5′-GATCAGCACACAGGTGATAATGAGG-3′

Areg-F: 5′-CCATCATCCTCGCAGCTATT-3′

Areg-R: 5′-CTTGTCGAAGCCTCCTTCTT-3′

Atgl-F: 5′-GAC GGA GAG AAC GTC ATC ATA TC-3′

Atgl-R: 5′-CCA CAG TAC ACC GGG ATA AAT-3′

Hsl-F: 5′-CAT CAA CCA CTG TGA GGG TAA G-3′

Hsl-R: 5′-AAG GGA GGT GAG ATG GTA ACT-3′

Mgl-F: 5′- TTA TAT GGG TGG GTG GGT AAA G-3′

Mgl-R: 5′- GAG AGC CAA CTA GTC CTC AAT TA-3′

Gapdh-F: 5′- AAC AGC AAC TCC CAC TCT TC-3′

Gapdh-R: 5′- CCT GTT GCT GTA GCC GTA TT-3′

All measurements were performed in triplicate (minimum, n = 3).

### Immunoblot

The cell or tissue lysates were collected using the RIPA buffer (Boston Bio-Products; MA) supplemented with Protease and Phosphatase Inhibitors (Thermo Scientific; MA). Briefly, sample proteins (30 or 40 µg) were separated by SDS-PAGE electrophoresis and then transferred to PVDF membranes (EMDMillipore; MA). After blocking with 5% BSA or non-fat milk for 1 h, the membranes were incubated with primary antibody overnight at 4 °C; The next day, the membranes were incubated with anti-rabbit, rat or mouse secondary antibody (Bio-Rad; CA) for 1 h; and the final detection was performed using the ECL Plus Western Blotting Detection Reagents (GE Healthcare; PA). We used the following antibodies: anti-Flag M2 antibody (Sigma-Aldrich; MO); anti-AKT, anti-pAKT (S473), anti-ATGL, anti-HSL, anti-ERK and anti-pERK antibodies (Cell Signaling Technology; MA); anti-GAPDH antibody (Ubiquitin-Proteasome Biotechnologies; CO).

### Immunohistochemical staining

Slides from formalin-fixed, paraffin-embedded tissues were first deparaffinized and rehydrated by incubations in the following solutions for 5 minutes each: Xylene 3 times, 100% EtOH 3 times, 95% EtOH once, 70% EtOH once, 50% EtOH once, and ddH_2_O once. Antigen unmasking was carried out in 10 mM citrate buffer (pH 6) using the microwave heating method for 10 minutes. Slides were allowed to cool at room temperature for 20 minutes, then under running water for 10 minutes. Slides were incubated in 3% H_2_O_2_ for 10 minutes, then washed under running water for 5 minutes. Slides were washed again by incubating in 1X TBST twice for 5 minutes. The tissue on each slide was circled using a hydrophobic pen (Vector Laboratories; CA) and blocked for 1 hour in 300 µl 1%BSA/PBS in a humidified chamber. Slides were then incubated in a humidified chamber overnight at 4 °C with 300ul of primary antibody in 1% BSA/PBS. On the following day, primary antibodies were removed and slides were washed in 1X TBST for 5 minutes, 3 times. Three drops of secondary antibody (Immpress HRP polymer reagents- Vector Laboratories, CA) were added to each slide and incubated for 1 hour at room temperature in a humidified chamber. Slides were washed in 1X TBST 3 times for 5 minutes. One hundred and fifty microliters DAB (Sigma) was added to each slide and incubated until section began to develop. Slides were immersed in water to stop reaction and washed in water twice for 5 minutes. Next, slides were counterstained in hematoxylin for 2.5 minutes and washed under running water for 5 minutes. Slides were dipped twice in acid/alcohol solution (75% EtOH + 25% HCl) and washed again under running water for 10 minutes. Slides were rehydrated by incubations in the following solutions for 5 minutes each: 50% EtOH once, 70% EtOH once, 95% EtOH once, 100% EtOH 3 times and Xylene 3 times. Permount was used to coverslip each slide. We used the following antibodies: anti-TAZ antibody (Sigma-Aldrich; MO); anti-Ki67 antibody (mouse preferred; IHC formulated; Cell Signaling Technology; MA).

### Statistical analysis

All statistical analysis of terminal end buds count, mammary gland weight, RT-PCR, circulating triglyceride release, glycerol release was performed with two-tailed Student’s t-tests; data are expressed as mean ± SD.

## Electronic supplementary material

Supplemental information

## References

[1] Pan D (2010). The hippo signaling pathway in development and cancer. Developmental cell.

[2] Harvey K, Tapon N (2007). The Salvador-Warts-Hippo pathway - an emerging tumour-suppressor network. Nature reviews. Cancer.

[3] Moroishi T, Hansen CG, Guan KL (2015). The emerging roles of YAP and TAZ in cancer. Nature reviews. Cancer.

[4] Harvey KF, Zhang X, Thomas DM (2013). The Hippo pathway and human cancer. Nature reviews. Cancer.

[5] Johnson R, Halder G (2014). The two faces of Hippo: targeting the Hippo pathway for regenerative medicine and cancer treatment. Nature reviews. Drug discovery.

[6] Bossuyt W (2014). An evolutionary shift in the regulation of the Hippo pathway between mice and flies. Oncogene.

[7] Yu FX (2012). Regulation of the Hippo-YAP pathway by G-protein-coupled receptor signaling. Cell.

[8] Chen D (2012). LIFR is a breast cancer metastasis suppressor upstream of the Hippo-YAP pathway and a prognostic marker. Nature medicine.

[9] Calvo F (2013). Mechanotransduction and YAP-dependent matrix remodelling is required for the generation and maintenance of cancer-associated fibroblasts. Nature cell biology.

[10] Aragona M (2013). A mechanical checkpoint controls multicellular growth through YAP/TAZ regulation by actin-processing factors. Cell.

[11] Dupont S (2011). Role of YAP/TAZ in mechanotransduction. Nature.

[12] Zhao B (2012). Cell detachment activates the Hippo pathway via cytoskeleton reorganization to induce anoikis. Genes & development.

[13] Inman JL, Robertson C, Mott JD, Bissell MJ (2015). Mammary gland development: cell fate specification, stem cells and the microenvironment. Development.

[14] Watson CJ, Khaled WT (2008). Mammary development in the embryo and adult: a journey of morphogenesis and commitment. Development.

[15] Brisken C, Rajaram RD (2006). Alveolar and lactogenic differentiation. Journal of mammary gland biology and neoplasia.

[16] Shi P, Feng J, Chen C (2015). Hippo pathway in mammary gland development and breast cancer. Acta biochimica et biophysica Sinica.

[17] Chen Q (2014). A temporal requirement for Hippo signaling in mammary gland differentiation, growth, and tumorigenesis. Genes & development.

[18] Skibinski A (2014). The Hippo transducer TAZ interacts with the SWI/SNF complex to regulate breast epithelial lineage commitment. Cell reports.

[19] Cordenonsi M (2011). The Hippo transducer TAZ confers cancer stem cell-related traits on breast cancer cells. Cell.

[20] Bartucci M (2015). TAZ is required for metastatic activity and chemoresistance of breast cancer stem cells. Oncogene.

[21] Frangou C (2014). Molecular profiling and computational network analysis of TAZ-mediated mammary tumorigenesis identifies actionable therapeutic targets. Oncotarget.

[22] Kim T (2015). A basal-like breast cancer-specific role for SRF-IL6 in YAP-induced cancer stemness. Nature communications.

[23] Lai D, Ho KC, Hao Y, Yang X (2011). Taxol resistance in breast cancer cells is mediated by the hippo pathway component TAZ and its downstream transcriptional targets Cyr61 and CTGF. Cancer research.

[24] Overholtzer M (2006). Transforming properties of YAP, a candidate oncogene on the chromosome 11q22 amplicon. Proceedings of the National Academy of Sciences of the United States of America.

[25] Lei QY (2008). TAZ promotes cell proliferation and epithelial-mesenchymal transition and is inhibited by the hippo pathway. Molecular and cellular biology.

[26] Li YW (2015). Characterization of TAZ domains important for the induction of breast cancer stem cell properties and tumorigenesis. Cell cycle.

[27] Moody SE (2002). Conditional activation of Neu in the mammary epithelium of transgenic mice results in reversible pulmonary metastasis. Cancer cell.

[28] Kitamura M (1998). Transgene regulation by the tetracycline-controlled transactivation system. Exp Nephrol.

[29] Whisenhunt TR (2006). Disrupting the enzyme complex regulating O-GlcNAcylation blocks signaling and development. Glycobiology.

[30] Ciarloni L, Mallepell S, Brisken C (2007). Amphiregulin is an essential mediator of estrogen receptor alpha function in mammary gland development. Proceedings of the National Academy of Sciences of the United States of America.

[31] Kenney NJ, Smith GH, Lawrence E, Barrett JC, Salomon DS (2001). Identification of Stem Cell Units in the Terminal End Bud and Duct of the Mouse Mammary Gland. Journal of biomedicine & biotechnology.

[32] Hinck L, Silberstein GB (2005). Key stages in mammary gland development: the mammary end bud as a motile organ. Breast cancer research: BCR.

[33] Yang N (2012). TAZ induces growth factor-independent proliferation through activation of EGFR ligand amphiregulin. Cell cycle.

[34] Hovey RC, Aimo L (2010). Diverse and active roles for adipocytes during mammary gland growth and function. Journal of mammary gland biology and neoplasia.

[35] Rutkowski JM, Stern JH, Scherer PE (2015). The cell biology of fat expansion. The Journal of cell biology.

[36] Bolsoni-Lopes A, Alonso-Vale MI (2015). Lipolysis and lipases in white adipose tissue - An update. Arch Endocrinol Metab.

[37] Fruhbeck G, Mendez-Gimenez L, Fernandez-Formoso JA, Fernandez S, Rodriguez A (2014). Regulation of adipocyte lipolysis. Nutr Res Rev.

[38] Zhou D (2009). Mst1 and Mst2 maintain hepatocyte quiescence and suppress hepatocellular carcinoma development through inactivation of the Yap1 oncogene. Cancer cell.

[39] Huang J, Wu S, Barrera J, Matthews K, Pan D (2005). The Hippo signaling pathway coordinately regulates cell proliferation and apoptosis by inactivating Yorkie, the Drosophila Homolog of YAP. Cell.

[40] Lee KP (2010). The Hippo-Salvador pathway restrains hepatic oval cell proliferation, liver size, and liver tumorigenesis. Proceedings of the National Academy of Sciences of the United States of America.

[41] Kanai F (2000). TAZ: a novel transcriptional co-activator regulated by interactions with 14-3-3 and PDZ domain proteins. The EMBO journal.

[42] Hong JH (2005). TAZ, a transcriptional modulator of mesenchymal stem cell differentiation. Science.

[43] Matsumoto Y (2016). Reciprocal stabilization of ABL and TAZ regulates osteoblastogenesis through transcription factor RUNX2. The Journal of clinical investigation.

[44] Park HW (2015). Alternative Wnt Signaling Activates YAP/TAZ. Cell.

[45] Huang H, Wu W, Zhang L, Liu XY (2013). Drosophila ste-20 family protein kinase, hippo, modulates fat cell proliferation. PloS one.

[46] Duncan RE, Ahmadian M, Jaworski K, Sarkadi-Nagy E, Sul HS (2007). Regulation of lipolysis in adipocytes. Annual review of nutrition.

[47] Wang S (2008). Lipolysis and the integrated physiology of lipid energy metabolism. Molecular genetics and metabolism.

[48] Strassburger K, Tiebe M, Pinna F, Breuhahn K, Teleman AA (2012). Insulin/IGF signaling drives cell proliferation in part via Yorkie/YAP. Developmental biology.

[49] Moeller ME (2017). Warts Signaling Controls Organ and Body Growth through Regulation of Ecdysone. Current biology: CB.

[50] Wang C (2016). YAP/TAZ regulates the insulin signaling via IRS1/2 in endometrial cancer. American journal of cancer research.

[51] Tao L, Xiang D, Xie Y, Bronson RT, Li Z (2017). Induced p53 loss in mouse luminal cells causes clonal expansion and development of mammary tumours. Nature communications.

[52] Yu FX, Zhao B, Guan KL (2015). Hippo Pathway in Organ Size Control, Tissue Homeostasis, and Cancer. Cell.

[53] Zanconato F, Cordenonsi M, Piccolo S (2016). YAP/TAZ at the Roots of Cancer. Cancer cell.

[54] Parashurama N (2012). Remodeling of endogenous mammary epithelium by breast cancer stem cells. Stem cells.

[55] Wilson KE (2014). PTPN14 forms a complex with Kibra and LATS1 proteins and negatively regulates the YAP oncogenic function. The Journal of biological chemistry.

