# Supplementary material for: The Hippo Signaling Transducer TAZ Regulates Mammary Gland Morphogenesis and Carcinogen-induced Mammary Tumorigenesis
Source: Sci Rep. 2018 Apr 24;8:6449. doi: 10.1038/s41598-018-24712-5 (PMC5915420; doi:10.1038/s41598-018-24712-5)

The Hippo Signaling Transducer TAZ Regulates Mammary Gland Morphogenesis and Carcinogen-induced Mammary Tumorigenesis

Kayla E. Denson [1], Ashley L. Mussell [1], He Shen [1], Alexander Truskinovsky [2], Nuo Yang [3], Natesh Parashurama[4], Yanmin Chen [1], Costa Frangou [5], Fajun Yang [6], and Jianmin Zhang [1*]

1. Department of Cancer Genetics & Genomics,

2. Department of Pathology, Roswell Park Cancer Institute, Buffalo, NY 14263

3. Department of Anesthesiology,

4. Department of Chemical & Biological Engineering, University at Buffalo, The State University of New York, NY 14214

5. Harvard TH Chan School of Public Health, Molecular and Integrative Physiological Sciences, 665 Huntington Avenue, Boston, MA 02115

6. Departments of Medicine, Diabetes Research Center, Albert Einstein College of Medicine, Bronx, NY 10461

*Corresponding author.

Running Title: TAZ Regulates Mammary Gland Morphogenesis

Key words: Hippo pathway, mammary gland, TAZ, organ size, tumorigenesis

Supplemental Figure legends

A. Schematic illustration of the TRE-TAZ^{4SA} construct.

B. TAZ induction by Dox-containing chow in the MMTV-rtTA and TRE-TAZ^{4SA} crossed mice.

C. Immunoblot of AKT, phospho-AKT (S473), ERK1/2 and phospho-ERK1/2 (Thr202/Tyr204) expression in WT or MMTV-rtTA/TRE-TAZ mammary gland. n=3 WT mice 3 mammary glands; n=3 MMTV-rtTA/TRE-TAZ mice 6 mammary glands.

Fig S1

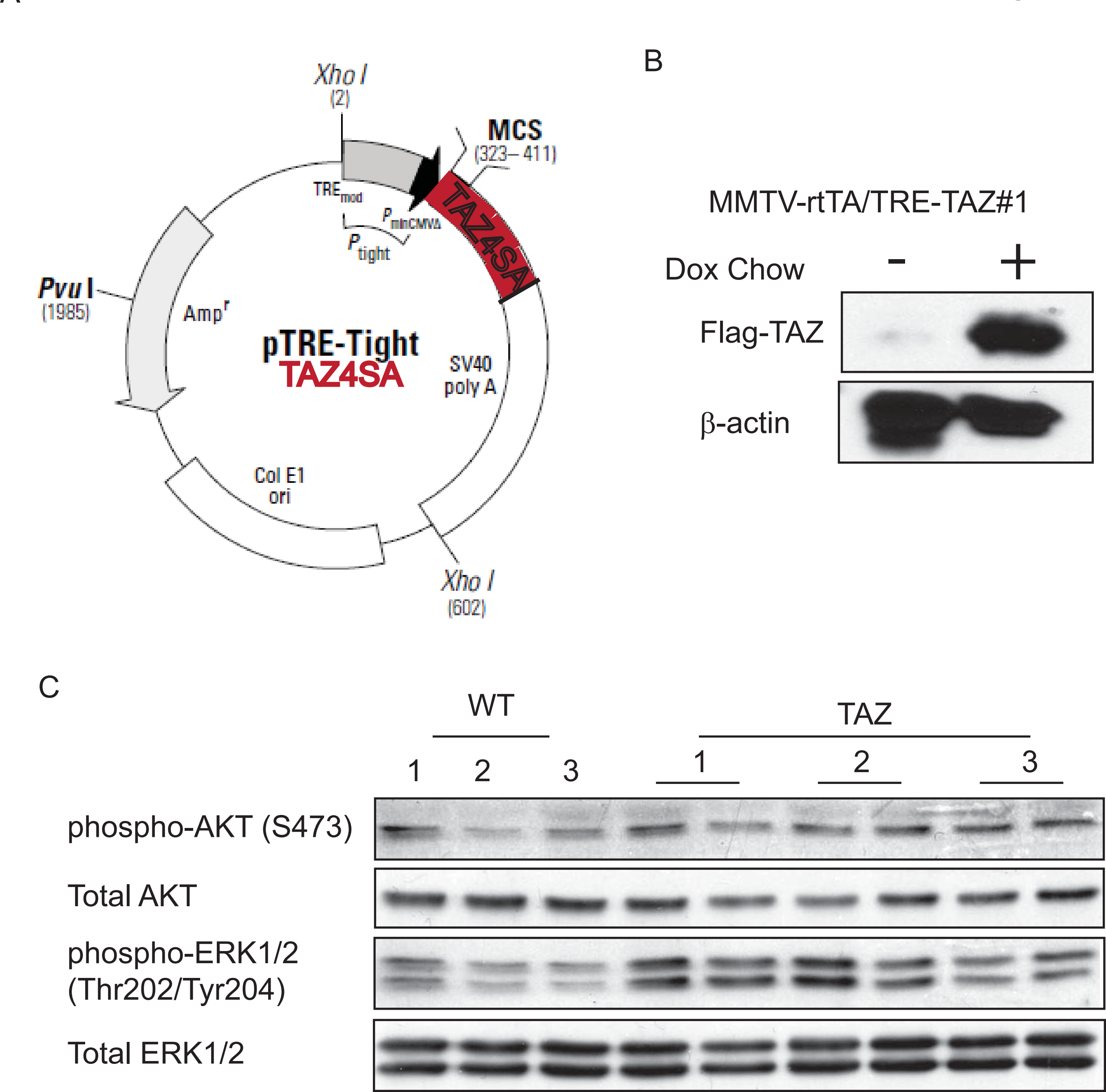

A
Xho I
(2)
MCS
(323– 411)
TREmod
PminCMVΔ
Ptight
TAZ4SA
Pvu I
(1985)
Ampr
pTRE-Tight
TAZ4SA
SV40
poly A
Col E1
ori
Xho I
(602)
B
MMTV-rtTA/TRE-TAZ#1
Dox Chow
-
+
Flag-TAZ
β-actin
C
WT
TAZ
1
2
3
1
2
3
phospho-AKT (S473)
Total AKT
phospho-ERK1/2
(Thr202/Tyr204)
Total ERK1/2

Uncropped images of all the western blot data

Figure1E

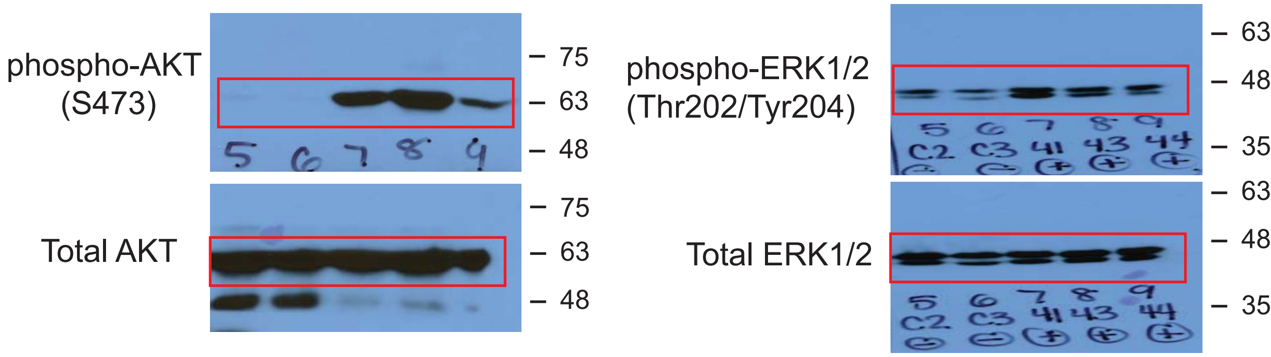

Figure3F

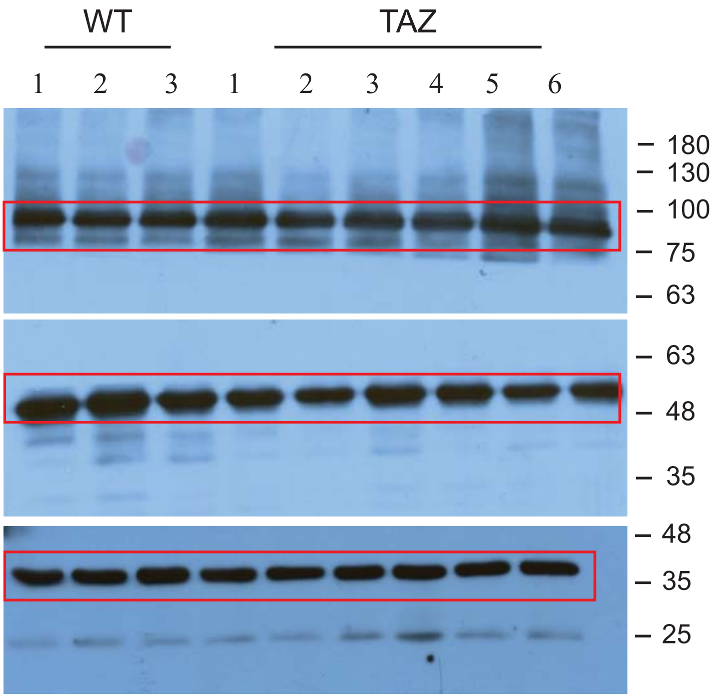

Figure 4F

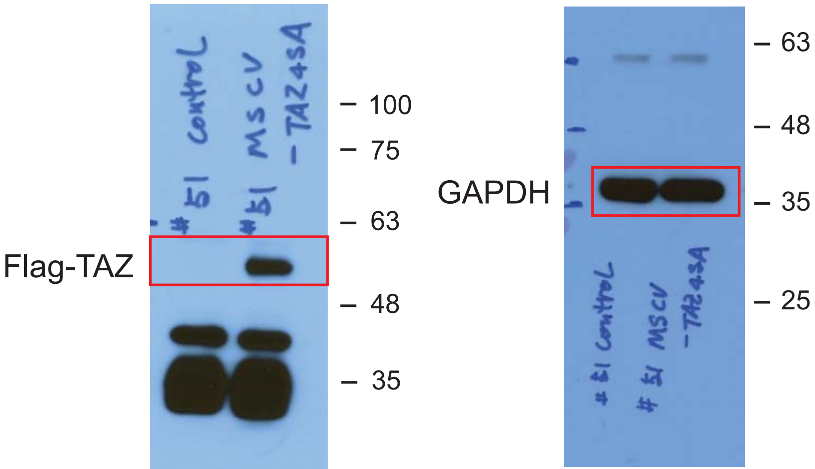

Uncropped images of all the western blot data (continued)

Figure S1B

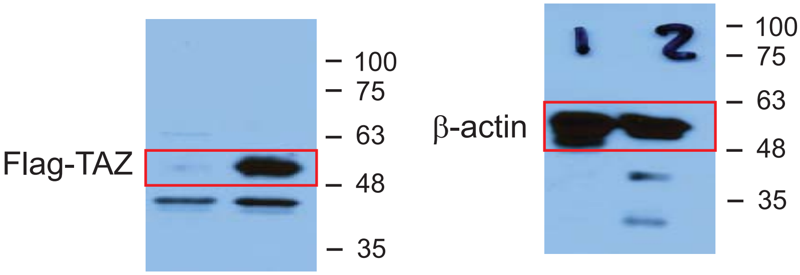

FigureS2

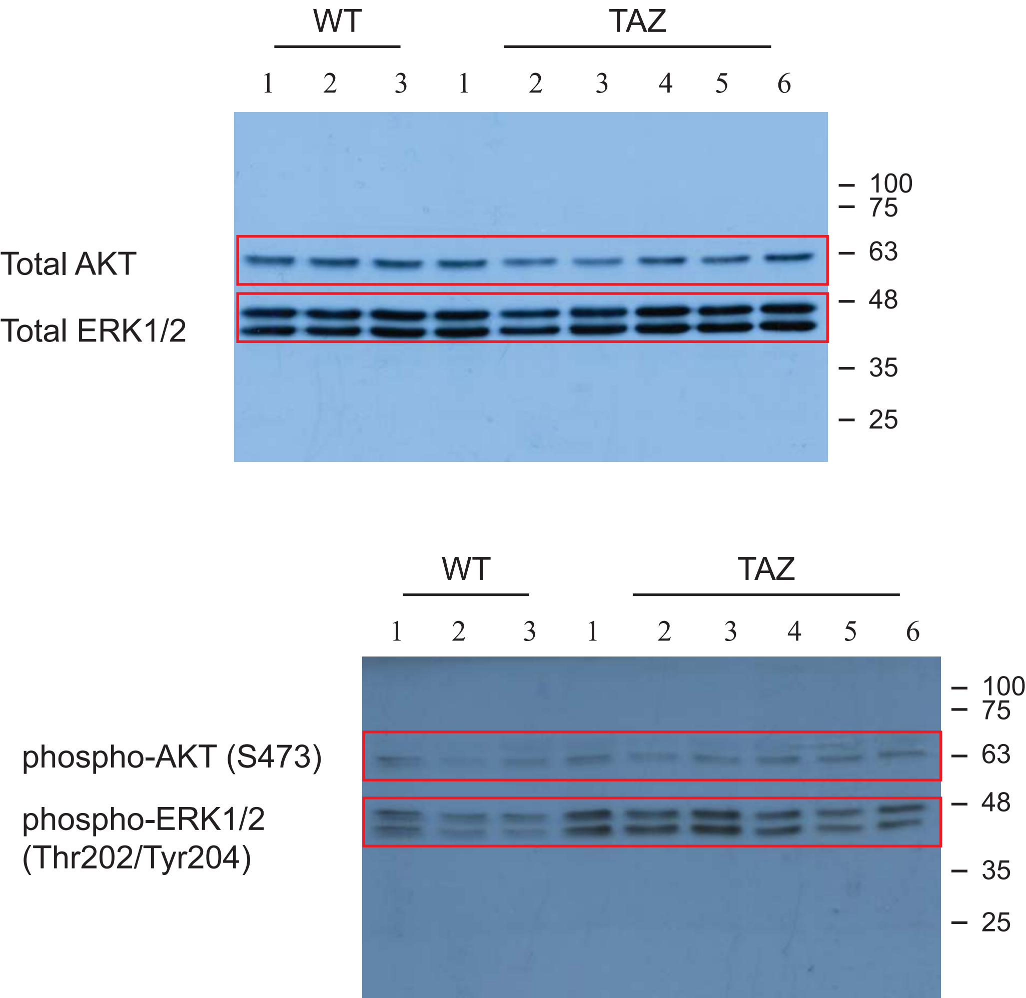

Supplement: Supplementary file 1 — Supplemental information [file 41598_2018_24712_MOESM1_ESM.pdf]
